# Prevalence of Tinea capitis in school going children from Mathare, informal settlement in Nairobi, Kenya

**DOI:** 10.1186/s13104-015-1240-7

**Published:** 2015-06-27

**Authors:** Jedidah Ndunge Moto, John Muthini Maingi, Anthony Kebira Nyamache

**Affiliations:** Department of Microbiology, Kenyatta University, Nairobi, Kenya

**Keywords:** Tinea capitis, Prevalence, Children, Dermatophytes, Informal settlement

## Abstract

**Background:**

Tinea capitis is a common infection especially in poor resource settings. This study was aimed at determining the prevalence Tinea capitis in children from selected schools from an urban slum in Nairobi city of Kenya.

**Methods:**

A cross-sectional study was carried out in 150 school going children during the period between May and September 2013. A questionnaire was administered and cultures of scalps, skin scrapping/hair stubs samples were performed and the etiological agents identified and confirmed.

**Results:**

In a total of one hundred and fifty (150) children recruited 89 (59.3%) were males and 61 (40.7%) females aged between 3 and 14 years. The overall prevalence rates in dermatophytes infection was 81.3% (122/150) with etiological agents consisting *Trichophyton* spp. (61.3%), *Microsporum* spp. (13.3%) and *Epidermophyton* spp. (7.3%) infections with infections occurring either singly (56%), duo (38%) or tipple co-infections (6%).

**Conclusion:**

This study demonstrates a high prevalence of Tinea infections with *Trichophyton tonsurans* as the predominant etiological agent in school going children of the urban slums of Nairobi.

**Electronic supplementary material:**

The online version of this article (doi:10.1186/s13104-015-1240-7) contains supplementary material, which is available to authorized users.

## Background

Tinea capitis is a common superficial fungal infection of the scalp and hair [1, 2]. This an exogenous infection that is characterized by invasion of dermatophytes into hair follicles and keratinized layer of hairy skin leading to hair loss, scaling, kerion, agminate folliculitis, favus, black dot, grey patch type, erythema or impetigo-like lesions [1]. The infection originates from diverse sources (human, animals and/or soil) [3] with high frequency occurring in prepubertal, school-going children aged between 6 and 10 years of age. Nevertheless, this infection occurs across age and sex groups with high occurrence being reported among males [3–6].

Tinea infections has remained a significant public health problem with poor hygiene, sharing of fomites, overcrowding and low socioeconomic being among some of the factors that predisposes populations to infections [4]. Despite high cases being detected in poor resource settings, these infections have a worldwide distribution with most cases being detected in Africa, Asia, and Southern and Eastern Europe [3, 7, 8]. Nevertheless, their epidemiological distribution has shown diverse geographical and seasonal variations depending on several factors, including life style, type of the population, migration of people and climatic conditions [9].

Tinea capitis is caused by a number of *Trichophyton,Microsporum* species [3, 10] and *Epidermophyton floccosum* of the genus *Epidermophyton* [10]. Worldwide, tinea infections are among the most common infectious agents in humans [8]. Its prevalence has continued to have a dramatic increase in the last decades with more than 20–25% of the world’s population being affected [11].

Africa, being among the settings mostly affected, the rates of tinea infection ranges between 10 and 30% among school-aged children [12]. Although *Tinea capitis*, like other dermatophytes, is of public health importance, it is not a notifiable disease and as a result, little is known on its prevalence in many endemic areas [4, 13–15]. Despite the elusive reports on the this infection in Kenya, 11.2% rates have been reported from Kibera slums, Nairobi. Nairobi, which is a capital city of Kenya, has a population of 3.1 million people [16] with more than half of them live in the informal settlements. These include; Kibera, Mathare slums, Korogocho slums and Mukuru Kwa Njenga slums all occupying 1% of the Nairobi area. Residents in these slums are faced with challenges of lack of food, poor housing, education and health. All these risks have put these slum dwellers on a high risk of contracting contagious diseases like tinea infections. [17] The aims of our study were to determine the prevalence, and causative agents of Tinea capitis among primary school going children in urban slums of Mathare in Nairobi, Kenya.

## Methods

This was a cross-sectional study consisting randomly recruited primary school going children from five public primary schools from Mathare, an informal settlement in Nairobi. Children aged between 4 and 14 years old consisting of 89 (59%) males and 61 (41%) females were enrolled into the study. This study was ethical approved by Kenyatta University Ethical approval committee before its execution. A questionnaire was administered to the subjects through consenting guardians and Scalp skin scrapping/hair stubs samples collected during period of May and September 2013.

Scalp skin scrapping/hair stubs were treated for 15–30 min with 1–2 drops of 20% KOH before being examined microscopically for fungal hyphae. Fungal cultures were done on Malt extract agar and considered positive if growth on day 14 or 28 and negative if there was no growth on day 28. Fungal identification was based on macroscopic (growth characteristics, pigment formation) and microscopic morphology (formation of macro conidia and micro conidia or other typical elements) [18]. In addition, urease test was also used in differentiating between some members of Trichophyton species [19].

### Data analysis

All the field data were collected and stored in Microsoft Excel Software package. The scalp scrapings/hair stubs data and questionnaire data were then exported to SPSS 16 for analysis. The results were then presented in descriptive statistics using frequency tables, cross tabulation and bar charts. Chi-square test was used for comparing prevalence of the infection by sex and age. P < 0.05 was considered significant.

## Results

### Socio demographic characteristics of study subjects

A total of one hundred ninety (190) children were invited to take part in the survey with only one hundred and fifty (150) (78.9%) of the participants’ guardians gave their consent to participate in the study. They comprised of 89 males and 61 females aged between 3 and 14 years with mean age of 8.5 ± 1.86 years all residing in Mathare slum in Nairobi. Majority of these children (76%) had more than 9 siblings in their family with those with less than four siblings being the least (2%). Majority of the parents of these children were unemployed 65/150 (43.3%) with those retired 24 (16%) being the least. Income of most of the parents was below Ksh 5,000 (54.7%) with least of them earning above Ksh. 20,000 (0.7%). With most of these children coming from poor settings, most of them 138/150 (92%) confirmed shairing items especially bath towels. Low economic status and unemployment were therefore found to be highly associated with high risk to tinea infections (*P* > 0.001) (Additional file 1: Table S1).

### Prevalence of *Tinea capitis* infection

In a total of 150 children examined, 122 (81.3%) were found to have fungal lesions caused by either one of three dermatophytes; *Trichophyton* (61.3%), *Microsporum* (13.3%) and *Epidermophyton* (7.3%). The symptomatic cases were 102 (68%) with 48 (32%) being asymptomatic. All symptomatic cases were confirmed positive while among the asymptomatic cases only 21 (43.75%) were confirmed positive for *Tinea infections*. According to gender, males (45.3%) were most infected compared to females (36.7%) with sex being a significant risk factor to infection (*P* = 0.020). However, across age groups, there was no significant difference in rates of tinea infections (*P* = 0.006) (Additional file 2: Table S2).

### Prevalence of dermatophytes

Of the total 150 samples examined, 11 (7.3%) were positive for *Epidermophyton* infections with children of the age group 6–8 years and males being the most affected (*P* = 0.330). The highest prevalence of *Microsporum* species infection was found in the age category 9–11 years followed by age group 6–8 years with those in ages groups 12–14 and 3–5 years being least affected. In the age category 9–11 years, males had significantly higher infections than females (*P* = 0.032) (Additional file 1: Table S1). However, *E. floccosum* was the only detected. Trichophyton species were the most detected 92 (61.3%) species in this study with *Trichophyton tonsurans* (45.3%) being the most prevalence and *T. verucosum* (4.3%) and *Trichophyton rubrum* (4.3%) being the least (Additional file 2: Table S2).

### Coinfections of diverse dermatophytes

Despite majority of the infections were caused a single etiological agents (56%), multiple infections (38%) were also detected. *Trichophyton* and *Microsporum* species co-occurred most at the rate of 30%, followed by *Trichophyton* and *Epidermophyton* combination at the rate of 6% and the least occurring combination was *Microsporum* species with *Epidermophyton* species at the rate of 2%. Triple infections with all the three dermatological agents observed in this study occurred at a very low rate of 6% (Additional file 3: Table S3).

## Discussion

Most studies have reported a significant burden of skin diseases among school going children. However, most of these studies have been conducted in rural areas. However, there are very few studies or none among school going children from urban slums. In this study we evaluated dermatological infections burden among school going children from informal settlement. Findings from this study revealed an overall prevalence of 81.2% (122/150). This rate was found to be higher than those obtained in Ethiopian (59%) [20], Tanzania (4%), [21] and those previous conducted in Kenya (33.3%) [4, 12]. The high prevalence rates obtained in this study could be associated with high population growth hence frequent contact with infected individuals. In relation to gender, males were most infected with high rates probably being associated with haircuts, uncleaned barbers, shairing of combs and the heavy mingling with friends without conscious on personal hygiene Contrarly to girls [22–26]. The low prevalence rates in girls could be associated with the facts that girls especially of the age above 13 years weave their hair and high general personal hygiene [23]. Nevertheless, these findings were contrary to those obtained in Egypt and Nigeria where girls had high prevalence rates of infection than boys [15, 27].

We determined whether age had any significant influence to rates of infection. However, there was no difference across the age groups in relation to infection. However, those of age groups between 3–5 and 6–8 (77.8%) had highest prevalence. A finding that is similar to those obtained from previous studies in Kenya and Sub Saharan African countries (69–78%) [4, 19] (Additional file 1: Table S1). This is can be explained by poor hygiene at this age as well as the absence of saturated fatty acids that provide a natural protective mechanism against dermatophytes [12]. Findings from this study showed that (Epidermophyton species (6.7%); Microsporum species (13.3%) and Trichophyton species (61.3%) were endemic in the study area with varied prevalence, a trend that was similar to those previously observed in other tropical Africa countries [4, 28, 29].

*Trichophyton* species had the highest prevalence of 61.3%, with *T. tonsurans* (33.3%) being the most predominant dermatophyte due its anthropophilic nature and abundance among human carriers [30]. These findings were similar to those obtained from previous studies in Kenya [4] and Nigeria 29]. For *Trichophyton mentagrophytes* (6.7%), the findings were similar those previously conducted in other sub-Saharan countries of 7.3–15.7% [29, 30]. However, among this genus, *Trichophyton verrucosum* and *T. rubrum* (8%), were least isolated. These findings were similar to previous studies that have shown a lowly maintained prevalence trend of this species in Kenya and Nigeria ranging between 4 and 9% [3, 4]. The recorded low prevalence rates in *T. verrucosum* among the studied subjects could be due low or non interactions with cattle that are the reservoir for this dermatophyte [28]. *T. rubrum* was also detected with low rates being recorded like elsewhere in the world [31]. *Microsporum gypseum, Microsporum canis and E. floccosum* were isolated in this study similar to those obtained in Nigeria [32] and Libya [28, 31]. In addition, dermatophyte co-infections were also detected indicating possible existence of poor personal hygiene, suboptimal treatment, poor drug adherence or abject poverty as some of the predisposing factors to tinea infections [33, 34].

## Conclusion

This study demonstrated a high prevalence of *Tineacapitis* with *T. tonsurans* as the most predominant etiological agent. In addition Illiteracy, Poverty and shairing of fomites were associated with high risk to Tinea infections. People living in this environment should be educated on how to practice personal and community hygiene, avoid sharing of items and improvement of household and environment hygiene. There is a need to improve living standards of Mathare residents. A need to check on antiseptic procedures used by the barbers and advice them on the correct procedures to be implemented. Therefore is there a need to have a regular epidemiological surveillance of causative fungal organisms in a community an essential component in the management of this condition.

## Additional files

Additional file 1:
**Table S1.** Tinea infections frequencies cases and socio—demographic characteristics of school going children. Additional file 2:
**Table S2.** Prevalence of *Tinea capitis* infections among the study subjects in Mathare informal settlement. Additional file 3:
**Table S3.** Dermatophytes frequency among school going children from urban slum.
