# Supplementary material for: Prevalence of Tinea capitis in school going children from Mathare, informal settlement in Nairobi, Kenya
Source: BMC Res Notes. 2015 Jun 27;8:274. doi: 10.1186/s13104-015-1240-7 (PMC4483201; doi:10.1186/s13104-015-1240-7)
Supplement: Additional file 1: — Table S1. Tinea infections frequencies cases and socio—demographic characteristics of school going children. [file 13104_2015_1240_MOESM1_ESM.pdf]

Table 1: Tinea infections frequencies cases and socio—demographic characteristics of school going children.

	n(150)	Tinea infections rates n (81.3%)	*P*=values
**Parents Occupation**			
Employed	34	9(26.5%)	*P*<0.001
Self-employed	27	19(70.4%)	
Unemployed	75	75(50%)	
Retired	33	28 (84.8%)	
**Subtotal**	150		
Male	89	(59.3%)	*p* =0.020)
Female	61	(40.7%)	
**Monthly income**			*P*<0.001
Below 5000	82	54.7	
5000-10,000	33	22	
10,000-15,000	21	14	
15,000-20,000	13	8.7	
Above 20,000	1	0.7	
**Number of children /family**			*P*=0.21
1-4	3	2	
5-8	33	22	
9-12	114	76	
Access to	81	73.6	*P*=0.191
NO access	29	26.4	
**Share of fomites/items**			
Yes	138	92	*P*<0.001
no	12	8	
Shared items			
combs	44	64	*P*<001
Bathing towels	52	28	
