# Supplementary material for: Prevalence of Tinea capitis in school going children from Mathare, informal settlement in Nairobi, Kenya
Source: BMC Res Notes. 2015 Jun 27;8:274. doi: 10.1186/s13104-015-1240-7 (PMC4483201; doi:10.1186/s13104-015-1240-7)
Supplement: Additional file 2: — Table S2. Prevalence of Tinea capitis infections among the study subjects in Mathare informal settlement. [file 13104_2015_1240_MOESM2_ESM.pdf]

Table 2: Prevalence of *Tinea capitis* infections among the study subjects in Mathare informal settlement

Age (yrs)	Sex Examined	Number (N)	*Trichophyton* species n (%)	*Microsporum* species n (%)	*Epidermophyton* species n (%)	overall
3-5	Male	24	22(91.6)*	1(4.2)	0(0)	23(95.8)
	Female	23	15(65.2)	4(17.4)	1(4.3)	20(87.0)
	Total	47	37(78.7)	5(10.6)	1(2.1)	43(91.4)
6-8	Male	42	26(61.9)	3(7.1)	5(11.9)	34(81.0)
	Female	30	14(46.7)	6(20.0)	2(6.7)	22(73.3)
	Total	72	40(55.6)	9(12.5)*	7(9.7)	56(77.8)*
9-11	Male	7	4(57.1)	3(42.9)	0(0)	7(100)*
	Female	10	6(60.0)	1(10.0)	2(20)	9(90)
	Total	17	10(58.8)*	4(23.5)	29(11.8)	16(94.10)
12-14	Male	6	4(66.7)	0(0)	1(16.7)	5(83.3)
	Female	3	1(33.3)	2(66.7)	0(0)	3(100)
	Total	9	5(55.6)	2(22.2)	1(11.1)	8(88.9)
All	Male	89	56(62.9)*	7(7.9)*	5(5.6)	68(76.4)*
	Female	61	36(59.0)	13(21.3)	6(9.8)	55(90.2)
	**Total**	**150**	**92(61.3)**	**20(13.3)**	**11(7.3)**	**122(81.3.0)**
