# Supplementary material for: Prevalence of Tinea capitis in school going children from Mathare, informal settlement in Nairobi, Kenya
Source: BMC Res Notes. 2015 Jun 27;8:274. doi: 10.1186/s13104-015-1240-7 (PMC4483201; doi:10.1186/s13104-015-1240-7)
Supplement: Additional file 3: — Table S3. Dermatophytes frequency among school going children from urban slum. [file 13104_2015_1240_MOESM3_ESM.pdf]

Table 3: Dermatophytes frequency among school going children from urban slum

Genus	Species	n	%	*P*=value
Microsporum	*canis*	9	6.0	p=0.330).
	Gypseum	11	7.3	
	Sub total	20	13.3	
Epidermorphyton	*floccosum*	10	6.7	
	Sub-total	10	6.7	
Trichophyton	*tonsurans*	68	45.3	
	mentagrophytes	10	6.7	
	verrucosum	7	4.3	
	rubrum	7	4.3	
	**Sub-total**	92	61.3	
Coinfections		57	38	
Mono-infections		84	56	
Duo infections	*Trichophyton/Microsporum*	45	30	
	Trichophyton/Epidermophyton	9	6	
Triple infections	*Trichophyton/Microsporum/Epidermophyton*	9	6	
